# Supplementary material for: Development and validation of an early risk-stratification model for hemophagocytic lymphohistiocytosis in severe fever with thrombocytopenia syndrome
Source: PLoS Negl Trop Dis. 2026 Apr 17;20(4):e0014247. doi: 10.1371/journal.pntd.0014247 (PMC13108874; doi:10.1371/journal.pntd.0014247)
Supplement: S1 Table — The table reports median (IQR), range, and the proportion meeting the HScore threshold (≥170) for each group. (DOCX) [file pntd.0014247.s001.docx]

**Table S1. HScore distributions within 3 days after virologic diagnosis and over the entire clinical course, stratified by final outcome group.**

| **Group** | **n** | **3-day HScore Median (IQR)** | **3-day HScore Range** | **3-day HScore >=170, n (%)** | **Entire-course HScore Median (IQR)** | **Entire-course HScore Range** | **Entire-course HScore >=170, n (%)** |
| --- | --- | --- | --- | --- | --- | --- | --- |
| Overall | 249 | 150.0 (120.0–185.0) | 43.0–259.0 | 83 (33.3%) | 156.0 (136.0–187.0) | 43.0–259.0 | 97 (39.0%) |
| Non-HLH (status=0) | 152 | 124.5 (106.0–150.0) | 43.0–167.0 | 0 (0.0%) | 150.0 (119.2–150.0) | 43.0–167.0 | 0 (0.0%) |
| HLH (status=1) | 97 | 191.0 (180.0–205.0) | 106.0–259.0 | 83 (85.6%) | 200.0 (185.0–220.0) | 170.0–259.0 | 97 (100.0%) |

*Notes: Values are median (IQR) and range unless otherwise indicated. HScore was calculated using variables available within 3 days after virological diagnosis and over the entire clinical course, respectively. The final outcome grouping (HLH vs non-HLH) in this study was based on the entire-course HScore threshold (≥170).*
