# Supplementary material for: Development and validation of an early risk-stratification model for hemophagocytic lymphohistiocytosis in severe fever with thrombocytopenia syndrome
Source: PLoS Negl Trop Dis. 2026 Apr 17;20(4):e0014247. doi: 10.1371/journal.pntd.0014247 (PMC13108874; doi:10.1371/journal.pntd.0014247)
Supplement: S3 Table — Results are presented as n/N (%) using evaluable denominators and document non-evaluable tests; HLH-2004 criteria are reported for descriptive reference only and were not used for binary outcome classification. (DOCX) [file pntd.0014247.s003.docx]

**Table S3. Fulfillment of individual HLH-2004 criteria over the entire clinical course, by outcome group.**

| **Criterion No.** | **Criterion** | **Non-HLH (status=0) - Met, n/N (%)** | **Non-HLH (status=0) - Not evaluable, n** | **HLH (status=1) - Met, n/N (%)** | **HLH (status=1) - Not evaluable, n** |
| --- | --- | --- | --- | --- | --- |
| 1 | Fever >38.5°C for >7 days | 83/152 (54.6%) | 0 | 72/97 (74.2%) | 0 |
| 2 | Splenomegaly | 3/72 (4.2%) | 80 | 11/67 (16.4%) | 30 |
| 3 | Cytopenias (>=2 cell lines) | 67/152 (44.1%) | 0 | 44/97 (45.4%) | 0 |
| 4 | Hypertriglyceridemia (>3 mmol/L) and/or hypofibrinogenemia (<1.5 g/L) | 39/152 (25.7%) | 0 | 64/97 (66.0%) | 0 |
| 5 | Hemophagocytosis in bone marrow/spleen/liver/lymph nodes | 0/9 (0.0%) | 143 | 7/11 (63.6%) | 86 |
| 6 | Low or absent NK-cell activity | Not assessed (N=0) | 152 | Not assessed (N=0) | 97 |
| 7 | Ferritin >=500 μg/L | 107/152 (70.4%) | 0 | 97/97 (100.0%) | 0 |
| 8 | Elevated soluble CD25 (sIL-2R) | Not assessed (N=0) | 152 | Not assessed (N=0) | 97 |

*Notes: Values are n/N (%) where N denotes the number of evaluable patients (criterion recorded as 0 or 1). Entries marked as 'N' or blank in the source data indicate tests not performed and are treated as not evaluable. NK-cell activity (criterion 6) and soluble CD25 (criterion 8) were not assessed in this cohort (0/249 evaluable). Hemophagocytosis (criterion 5) was evaluated only in patients who underwent tissue/bone marrow assessment; denominators therefore reflect this subset.*
