# Supplementary material for: Development and validation of an early risk-stratification model for hemophagocytic lymphohistiocytosis in severe fever with thrombocytopenia syndrome
Source: PLoS Negl Trop Dis. 2026 Apr 17;20(4):e0014247. doi: 10.1371/journal.pntd.0014247 (PMC13108874; doi:10.1371/journal.pntd.0014247)
Supplement: S1 Text — Transparent Reporting of a multivariable prediction model for Individual Prognosis Or Diagnosis (TRIPOD) checklist for the development and validation of an early risk-stratification model for hemophagocytic lymphohistiocytosis in severe fever with thrombocytopenia syndrome. BMJ 2024; 385 doi: https://doi.org/10.1136/bmj-2023-078378 (Published 16 April 2024). (DOCX) [file pntd.0014247.s007.docx]

**TRIPOD Checklist Template (2015)***Transparent Reporting of a multivariable prediction model for Individual Prognosis Or Diagnosis*

Generated: 2025-11-22

Purpose: Self-audit checklist for studies on development (D), external validation (V), or both (D;V) of multivariable prediction models. Tick "□" and indicate the manuscript location (page/line/paragraph).

| Manuscript title | Development and Validation of an Early Risk Prediction Model for HLH in SFTS | Corresponding author | Zhu,Chuan-Long |
| --- | --- | --- | --- |
| Model type (diagnostic/prognostic) | prognostic | Study type (development/validation/updating) | development |
| Version/date | 2025-11-22 | Guideline | TRIPOD 2015 (22 items) |

Applicability codes: D = Development; V = Validation; D;V = Applicable to both.

| Section | Item | Applicability (D/V/D;V) | Checklist item | Manuscript location / Done □ |
| --- | --- | --- | --- | --- |
| Title & abstract | 1 | D;V | Title: Identify the study as development and/or validation of a prediction model, including the target population and outcome. | □ Lines 1 |
| Title & abstract | 2 | D;V | Abstract: Structured summary of objectives, study design, setting, participants, sample size, predictors, outcome, statistical analysis, results, and conclusions (for validation: report performance measures). | □ Lines 24-52 |
| Introduction | 3a | D;V | Background: Explain the medical context (including any existing models) and the rationale for developing or validating the model. | □ Lines 61-77 |
| Introduction | 3b | D;V | Objectives: Specify whether the study concerns development, validation, or updating of a diagnostic or prognostic model. | □ Lines 78-82 |
| Methods | 4a | D;V | Source of data and study design: e.g., randomized trial, cohort, registry; single- or multicenter; how data were collected. | □ Lines 85-91 |
| Methods | 4b | D;V | Key study dates: start and end of accrual; end of follow-up if applicable. | □ Lines 86-91 |
| Methods | 5a | D;V | Study setting: primary/secondary/tertiary care or general population; number and location of centers. | □ Lines 85-91 |
| Methods | 5b | D;V | Eligibility criteria for participants. | □ Lines 93-103 |
| Methods | 5c | D;V | Treatments received, if relevant. | □ ________ |
| Methods | 6a | D;V | Outcome: define clearly what is being predicted, including how and when it was assessed. | □ Lines 98-103 |
| Methods | 6b | D;V | Report any actions taken to blind the assessment of the outcome. | □ ________ |
| Methods | 7a | D;V | Predictors: define all predictors and how and when they were measured. | □ Lines 105-118 |
| Methods | 7b | D;V | Report any actions taken to blind the assessment of predictors. | □ ________ |
| Methods | 8 | D;V | Sample size: explain how it was determined (e.g., number of events, events-per-parameter criteria). | □ Lines 86-91 |
| Methods | 9 | D;V | Missing data: describe how these were handled (complete-case analysis, single or multiple imputation) with details. | □ Lines 113-115 |
| Methods | 10a | D | Statistical analysis—handling of predictors: describe any transformations, categorization, coding (e.g., splines), or standardization. | □ Lines 116-118 |
| Methods | 10b | D | Model-building procedures: model type; predictor selection methods; internal validation procedures. | □ Lines 126-133 |
| Methods | 10c | V | For validation: describe how predictions (e.g., linear predictor) were calculated. | □ Lines 133-134 |
| Methods | 10d | D;V | Measures to assess model performance (e.g., discrimination, calibration) and, if relevant, to compare models. | □ Lines 134-137 |
| Methods | 10e | V | Describe any model updating (e.g., recalibration). | □ Lines 189-190, 197-198 |
| Methods | 11 | D;V | Risk groups: provide details on how risk groups were created, if done. | □ ________ |
| Methods | 12 | V | For validation: describe any differences from the development data (setting, eligibility, outcome, predictors, treatments). | □ Lines 89-91 |
| Results | 13a | D;V | Participant flow: numbers with and without the outcome; follow-up time; consider a flow diagram. | □ Lines 85-91, 93-103, 139-152, 474-480 |
| Results | 13b | D;V | Participant characteristics: demographics, clinical features, and predictor distributions; report numbers with missing data. | □ Lines 140-162, 398-427 |
| Results | 14a | D | Model development—numbers of participants and outcome events in each analysis. | □ Lines 102-103, 131-133, 183-184 |
| Results | 14b | D | If done, report unadjusted associations between candidate predictors and the outcome. | □ Lines 163-174 |
| Results | 15a | D | Present the full prediction model to allow individual predictions (all regression coefficients and intercept/baseline risk or survival). | □ Lines 175-179, 436-444 |
| Results | 15b | D | Explain how to use the prediction model in practice. | □ Lines 440-444 |
| Results | 16 | D;V | Report performance measures for the model, with confidence intervals. | □ Lines 180-199, 445-459 |
| Results | 17 | V | If done, report the results from model updating. | □ Lines 189-190, 197-198 |
| Discussion | 18 | D;V | Limitations: discuss potential sources of bias, sample size considerations, missing data, and other limitations. | □ Lines 246-258 |
| Discussion | 19a | D | Interpretation—development: provide an overall interpretation in the context of other evidence. | □ Lines 203-225 |
| Discussion | 19b | V | Interpretation—validation: compare with the development study and other validation studies and interpret differences. | □ Lines 226-236 |
| Discussion | 20 | D;V | Implications: discuss the potential clinical use of the model and implications for future research. | □ Lines 237-245, 259-269 |
| Other information | 21 | D;V | Supplementary information: protocol, data set, web calculator, and/or code availability. | □ Lines 288-290, 461-486 |
| Other information | 22 | D;V | Funding: report the source of funding and the role of the funders. | □ Lines 276-278 |

Note: Based on the official TRIPOD 2015 22-item checklist. Use with the Explanation & Elaboration document when preparing manuscripts.
