# Supplementary material for: Development and validation of an early risk-stratification model for hemophagocytic lymphohistiocytosis in severe fever with thrombocytopenia syndrome
Source: PLoS Negl Trop Dis. 2026 Apr 17;20(4):e0014247. doi: 10.1371/journal.pntd.0014247 (PMC13108874; doi:10.1371/journal.pntd.0014247)
Supplement: Attachment — Submitted filename: Response to Reviewers.docx [file pntd.0014247.s009.docx]

Dear Dr. Rothman and Editors,

Thank you for the editorial assessment of our manuscript, “Development and Validation of an Early Risk Prediction Model for HLH in SFTS.” We appreciate the feedback. We revised the manuscript to address all points raised prior to external peer review.

HLH status (binary outcome) is defined by the entire-course HScore >=170. We calculated HScore using the worst values over the clinical course.

Several HLH-2004 criteria were not routinely assessed. Some values are missing. Therefore, HLH-2004 criteria are reported for descriptive reference and transparent accounting only. They were not used to define the binary outcome.

Outcome accounting is provided in Supporting Information Tables S1 to S3.

Below are our point-by-point responses.

**Additional Editor Comments**

**Comment 1.** Please provide HScore summary statistics by group. Please report HLH-2004 criterion-level fulfillment and the total number of criteria met.

**Response:** We agree. We added complete outcome accounting.

- Table S1 reports HScore distributions by outcome group at two time windows: (i) within 3 days after virological confirmation and (ii) the entire course.
- Within 3 days: Non-HLH median 124.5 (IQR 106.0-150.0), range 43.0-167.0; HLH median 191.0 (IQR 180.0-205.0), range 106.0-259.0. Entire course: Non-HLH median 150.0 (IQR 119.25-150.0), range 43.0-167.0; HLH median 200.0 (IQR 185.0-220.0), range 170.0-259.0.
- Tables S2-S3 report HLH-2004 criteria as n/N (%), where N denotes evaluable patients for each criterion. “N” or blank indicates not performed and is treated as not evaluable; denominators therefore vary.
- Tables S2-S3 Notes summarize the total number of fulfilled criteria per patient and the number of evaluable criteria per patient (median [IQR] and range, by outcome group).

**Comment 2.** Please report early HScore and early HLH-2004 accounting at the same time point used for predictors.

**Response:** We agree. We aligned outcome accounting to the 3-day post-confirmation window.

- Early HScore (Table S1): Within 3 days, 83/249 (33.3%) met HScore >=170. In the final HLH group (entire-course HScore >=170; n=97), 83/97 (85.6%) met the threshold within 3 days. In the non-HLH group, 0/152 (0.0%) met the threshold within 3 days.
- Early HLH-2004 accounting (Table S2): Within 3 days, we report criterion-level fulfillment as n/N (%) and summarize fulfilled/evaluable criteria counts in the Table S2 Notes.

We clarified in the manuscript that “early” refers to the post-confirmation window used for model predictors.

**Comment 3.** Table 1 is unclear about the time window. Please clarify symptom definitions and max/min rules.

**Response:** We revised Table 1 and the Methods. Table 1 now reflects within 3 days after virological confirmation. We clarified symptom definitions. We state that labs use the most abnormal values within the 3-day window (max or min, as appropriate).

**Comment 4.** Figure 1: 95% confidence intervals are not visible.

**Response:** We updated Figure 1 to clearly display 95% confidence intervals for each AUC. The legend now matches the figure. We regenerated the figure files to meet PLOS technical requirements.

**Editorial Office Requirements**

**(1) CRediT author contributions incomplete.** Response: We completed CRediT contributions for all co-authors in the submission system under “Add/Edit/Remove Authors.”

**(2) Supporting Information legends missing.** Response: We added a Supporting Information legends section after the references. It lists Tables S1 to S3 and Figures S1 to S2.

Thank you again for your time and consideration. We hope the revision addresses the concerns and is ready for external peer review.

Sincerely,

Chuanlong Zhu (on behalf of all authors)

The First Affiliated Hospital with Nanjing University, Nanjing 210029, China.

Email: zhuchuanlong@jsph.org.cn
