# Supplementary material for: Development and validation of an early risk-stratification model for hemophagocytic lymphohistiocytosis in severe fever with thrombocytopenia syndrome
Source: PLoS Negl Trop Dis. 2026 Apr 17;20(4):e0014247. doi: 10.1371/journal.pntd.0014247 (PMC13108874; doi:10.1371/journal.pntd.0014247)
Supplement: Attachment — Submitted filename: Response_to_Reviewers_auresp_2.docx [file pntd.0014247.s010.docx]

**Response to the Editor and Reviewers**

Dear Dr. Rothman and Editors,

Thank you for the careful evaluation of our manuscript, **“**Development and Validation of an Early Risk-Stratification Model for Hemophagocytic Lymphohistiocytosis in Severe Fever with Thrombocytopenia Syndrome.**”** We sincerely appreciate the constructive comments from the Editor and Reviewers. We have revised the manuscript accordingly and believe that the revised version has been substantially improved.

Below we respond point by point.

**Editor comment**

**Comment:** The manuscript should explicitly acknowledge that a high percentage of HLH subjects already met HScore criteria at the early evaluation point, and that further study is needed to assess whether the model offers an advantage over HScore in timeliness of HLH identification.

**Response:**
We thank the Editor for this important point. We have revised the manuscript to frame the model more cautiously as an early risk-stratification tool rather than as proof of earlier identification than HScore. We now explicitly report that 83 of 97 patients (85.6%) in the final HLH group already met the HScore threshold at the early evaluation point. This point has been added to the Results, Discussion, and Limitations sections. We also now state clearly that the present study does not establish a timeliness advantage over HScore and that direct prospective comparisons are needed.

**Reviewer 1**

**Comment 1:** Please specify the duration of symptoms (median) when data were captured for model development and also initial HLH score calculation.

**Response:**
We have clarified the timing framework in the revised manuscript. The interval from symptom onset to admission was recorded and used as an operational summary of symptom duration at the time early data were captured for model development. In the overall cohort, this interval was 7 days (IQR, 5–8). We also clarified that both predictor ascertainment and descriptive early HScore accounting were anchored to the first 3 days after virologic confirmation, which constituted the predefined early-evaluation window.

**Comment 2:** About 85% of the HLH group already had HLH at the time initial data were collected, so this might not be an early prediction.

**Response:**
We agree and have revised the manuscript accordingly. Throughout the manuscript, we replaced stronger wording such as “early prediction” with more cautious language emphasizing early risk stratification. We now explicitly report that 83 of 97 patients (85.6%) in the final HLH group already met HScore criteria at the early evaluation point. We therefore clarify that the model is intended to support early risk recognition within a clinically relevant window, rather than to demonstrate superiority to HScore in timeliness.

**Reviewer 3**

**Comment:** Please spell out acronyms in the title.

**Response:**
We revised the title to spell out both disease names in full.

**Comment:** Please explain HScore so that the paper is more broadly accessible.

**Response:**
We added a brief explanation of HScore in the Introduction, including its purpose and key component domains, and clarified that it was not specifically developed for SFTS.

**Comment:** Is there data on time between symptom onset and positive test return?

**Response:**
We clarified this point in the Methods. Exact laboratory report-return timestamps were not consistently extractable from the retrospective record. However, in routine clinical practice, positive nucleic acid results were typically returned within approximately half a day of virologic confirmation. We therefore used the recorded time of virologic confirmation as the operational temporal anchor.

**Comment:** A figure showing differences between HLH and non-HLH patients would be useful.

**Response:**
We added Figure S3, which visually summarizes selected early clinical and laboratory differences between patients with and without HLH.

**Comment:** Figure 2 needs more context.

**Response:**
We revised the Figure 2 legend to clarify that the nomogram is intended for bedside early risk stratification during early hospital evaluation and added step-by-step usage instructions.

**Comment:** Figure S2 needs more explanation.

**Response:**
We revised the Figure S2 legend to explain the LASSO procedure and variable retention in more accessible language and clarified the role of cross-validation and Boruta confirmation.

**Comment:** Please discuss how this model could affect patient care trajectory.

**Response:**
We added a dedicated paragraph in the Discussion describing the likely clinical role of the model in early monitoring, repeated HLH assessment, earlier consultation, and earlier escalation of supportive care. We also clarified that the incremental value of the model may diminish once patients already meet established diagnostic criteria or have progressed to advanced organ failure.

**Comment:** How do your results compare with studies from Korea, Japan, and China?

**Response:**
We expanded the Discussion to compare our study more explicitly with prior studies from Korea, Japan, and China, and to highlight how the present study differs in its focus on HLH risk stratification, the use of six routinely available early variables, and the inclusion of external validation.

We sincerely thank the Editor and Reviewers for their thoughtful and constructive comments. We believe that the revised manuscript has been substantially strengthened and hope that it is now suitable for publication.

Sincerely,
Chuanlong Zhu (on behalf of all authors)
The First Affiliated Hospital with Nanjing Medical University, Nanjing 210029, China.

Email: zhuchuanlong@jsph.org.cn
